# Notes on three conjectures involving the digamma and generalized digamma functions

**DOI:** 10.1186/s13660-018-1936-z

**Published:** 2018-12-12

**Authors:** Ladislav Matejíčka

**Affiliations:** Faculty of Industrial Technologies in Púchov, Trenčín University of Alexander Dubček in Trenčín, Púchov, Slovakia

**Keywords:** 33B15, 26A48, 26A51, Digamma function, $(p, k)$-digamma function, Complete monotonicity, Monotonicity, Inequalities

## Abstract

In the paper, we solve one conjecture on an inequality involving digamma function, an open problem, and a conjecture on monotonicity of functions involving generalized digamma function. We also prove a new inequality for digamma function.

## Introduction

In the last years, the $(p,k)$-analogue of the gamma and polygamma functions has been studied intensively by a lot of authors. For historical background of the theory, see, for example, [1–24].

It is well known that:

- a function *f* is said to be completely monotonic [6, 21] on an interval *I* if *f* has derivatives of all orders on *I* and
  1 $$ (-1)^{n}f^{(n)}(x)\geq0 $$
  for $x\in I$, $n\geq0$, $n\in N$ (due to $0\in N$).
- the Euler gamma function [14–16, 20, 22, 23] is defined by
  2 $$ \varGamma(x)= \int_{0}^{\infty}t^{x-1}e^{-t} \,dt $$
  for $x>0$;
- the digamma function [11–13, 24] is defined by
  3 $$ \psi(x)=\frac{\varGamma'(x)}{\varGamma(x)}=-\gamma-\frac {1}{x}+\sum _{n=1}^{\infty}\frac{x}{n(n+x)}, $$
  where *γ* is the Euler–Mascheroni constant [5].

Recently, Díaz and Pariguan [4] defined the generalized gamma function

4 $$ \varGamma_{k}(x)=\lim_{n\rightarrow\infty}\frac {n!k^{n}(nk)^{\frac{x}{k}-1}}{x(x+k)\cdots (x+(n-1)k)} $$

for $k>0$ and $x\in C\setminus kZ^{-}$ and the generalized digamma function

5 $$ \psi_{k}(x)=\frac{\varGamma_{k}'(x)}{\varGamma _{k}(x)}=\frac{\ln(k)-\gamma}{k}-\frac{1}{x}+\sum _{n=1}^{\infty }\frac{x}{nk(nk+x)}. $$

Very recently, Nantomah, Prempeh, and Twum [8] introduced a new definition of the $(p,k)$-gamma function

6 $$ \varGamma_{pk}(x)=\frac{(p+1)!k^{p+1}(pk)^{\frac {x}{k}-1}}{x(x+k)\cdots (x+pk)} $$

for $k>0$ and $x>0$, $p\geq0$, $p\in N$, and the $(p,k)$-digamma function

7 $$ \psi_{pk}(x)=\frac{\varGamma_{pk}'(x)}{\varGamma _{pk}(x)}=\frac{\ln(pk)}{k}-\sum _{n=0}^{p}\frac{1}{nk+x} $$

for $k>0$ and $x>0$, $p\geq0$, $p\in N$.

We note that

$$\begin{aligned}& \lim_{k\rightarrow1}\psi_{k}(x) = \psi(x),\qquad \lim _{k\rightarrow 1}\varGamma_{k}(x)=\varGamma(x), \\& \lim_{p\rightarrow\infty}\varGamma_{pk}(x) = \varGamma_{k}(x), \qquad \lim_{p\rightarrow\infty}\psi_{pk}(x)=\psi_{k}(x). \end{aligned}$$

Li Yin, Li-Guo Huang, Zhi-Min Song, and Xiang Kai Dou [19] posed the following conjecture.

### Conjecture 1

([19])

*For* $p>0$ *and* $k\geq1$, *the function*

$$ \phi_{pk}(x)=\psi_{pk}(x)+\ln\bigl(e^{\frac{1}{x}-\frac {1}{x+pk+k}}-1\bigr) $$

*is strictly decreasing from* $(0,\infty)$ *onto* $(-\infty,\phi_{pk}(k))$.

Li Yin [17] posed the following open problem.

### Open Problem 1

([17])

If the function

$$ \delta_{pk\alpha}(x)=x^{\alpha} \biggl[\frac {1}{k}\ln \frac{pkx}{x+k(p+1)}-\psi_{pk}(x) \biggr] $$

is completely monotonic on $(0,\infty)$, then is it true that $\alpha \leq1$?

Yuming Chu, Xiaoming Zhang, and Xiaoming Tang [3] posed the following conjecture.

### Conjecture 2

*For* $b > a > 0$, *we have*

$$ \bigl(b-L(a, b)\bigr)\psi(b)+\bigl(L(a, b)-a\bigr)\psi(a)>(b-a)\psi (\sqrt{ba}), $$

*where* $L(a, b)=(b-a)/(\ln(b)-\ln(a))$.

The goal of the paper is to solve Conjecture 1, Conjecture 2, and Open Problem 1.

## Methods

In this paper, we use methods of mathematical and numerical analysis. We also use the software MATLAB for some computing.

## Results and discussion

In this section, we disprove Conjecture 1 (see [19]) and Conjecture 2 (see [3]) and prove one new inequality (Theorem 1) and Open Problem 1 (see [17]).

### Disproving Conjecture 1

It is evident that $\phi_{pk}(x)$ is strictly decreasing only if $e^{\phi_{pk}(x)}$ is strictly decreasing. We have

$$ e^{\phi_{pk}(x)}(x)=v_{pk}(x)=e^{\psi _{pk}(x+k)}-e^{\psi_{pk}(x)}. $$

Using Matlab, we obtain Table 1.

**Table 1:** Values of *p*, *k*, $x_{1}$, $x_{2}$, $v_{pk}(x_{1})$, $v_{pk}(x_{2})$

*p*	*k*	$x_{1}$	$v_{pk}(x_{1})$	$x_{2}$	$v_{pk}(x_{2})$
100,000	1.1	0.1	0.733034946365922	0.25	0.849008046429035
100,000	1.6	0.1	0.994429244720941	0.25	1.060815738973497
100,000	2.1	0.1	1.121247007413613	0.25	1.157039128396737
100,010	1.1	0.1	0.733034947426004	0.25	0.849008047776431
100,010	1.6	0.1	0.994429244720941	0.25	1.060815740083848
100,010	2.1	0.1	1.121247008239857	0.25	1.157039129298960

The table shows that $v_{pk}(x_{1})< v_{pk}(x_{2})$ for $0< x_{1}< x_{2}$, $p=100\text{,}000$, $p=100\text{,}010$, $k=1.1$, $k=1.6$, $k=2.1$. So $\phi_{pk}(x)$ is not strictly decreasing on $(0,\infty)$ for $p>0$ and $k>1$.

#### Remark 1

We note that Conjecture 1 (see [19]) is false since $\lim_{x\rightarrow0^{+}}v'_{pk}(x)>0$ for $p\geq1$ and $k>0$.

Indeed, differentiation of $v_{pk}(x)$ yields

$$ v'_{pk}(x)=e^{\psi_{pk}(x+k)}\psi'_{pk}(x+k)-e^{\psi _{pk}(x)} \psi'_{pk}(x). $$

Because of

$$\begin{aligned} \lim_{x\rightarrow0^{+}}e^{\psi_{pk}(x)}\psi '_{pk}(x) =& \lim_{x\rightarrow0^{+}} e^{\frac{\ln(pk)}{k}-\sum_{n=1}^{p}\frac{1}{nk+x}}e^{-\frac {1}{x}} \Biggl( \frac{1}{x^{2}}+\sum_{n=1}^{p} \frac {1}{(nk+x)^{2}} \Biggr) \\ =&C_{pk}\lim_{t\rightarrow+\infty}e^{-t}t^{2}=0, \end{aligned}$$

where $t=1/x$, and $C_{pk}>0$ is a constant, we obtain

$$ \lim_{x\rightarrow0^{+}}v'_{pk}(x)=e^{\psi _{pk}(k)} \psi'_{pk}(k)>0 $$

for $p\geq1$ and $k>0$. This implies that, for all $k>0$ and $p\geq 1$, there is $x_{pk}>0$ such that $v_{pk}(x)$ is a strictly increasing function on $(0,x_{pk})$. So, $\phi_{pk}(x)$ is a strictly increasing function on $(0,x_{pk})$.

Next, by the mean value theorem we get

$$\begin{aligned} v'_{pk}(x) =&e^{\psi_{pk}(x+k)}\psi '_{pk}(x+k)-e^{\psi_{pk}(x)} \psi'_{pk}(x) \\ =&e^{\psi_{pk}(\xi_{pkx})} \bigl(\psi_{pk}^{'2}( \xi_{pkx})+\psi ''_{pk}( \xi_{pkx}) \bigr)k=e^{\psi_{pk}(\xi_{pkx})}w_{pk}(\xi_{pkx})k, \end{aligned}$$

where $x<\xi_{pkx}<x+k$.

Due to

$$\begin{aligned} w_{pk}(x) =&\frac{1}{x^{4}} \Biggl[ \Biggl(1+\sum _{n=1}^{p}\frac{x^{2}}{(nk+x)^{2}} \Biggr)^{2} -2x \Biggl(1+\sum_{n=1}^{p} \frac{x^{3}}{(nk+x)^{3}} \Biggr) \Biggr] \\ < &\frac{1}{x^{4}} \bigl[(1+p)^{2}-2x \bigr], \end{aligned}$$

we obtain that, for all $k>0$ and $p\geq1$, the function $v'_{pk}(x)$ is a negative function on $((1+p)^{2}/2,+\infty)$. So, $\phi_{pk}(x)$ is a strictly decreasing function on $((1+p)^{2}/2,+\infty)$.

Finally, computer calculations show that, for $p\geq1$ and $k>1$, there is $0< x_{pk}<1$ such that $\phi_{pk}(x)$ is an increasing function on $(0,x_{pk})$ and a decreasing function on $(x_{pk},+\infty)$.

### Proof of Open Problem 1

Let $\delta_{pk\alpha}(x)$ be a completely monotonic function on $(0,\infty)$. Then $(-1)^{n}\delta^{(n)}_{pk\alpha}(x)\geq0$ for $x\in(0,\infty)$ and $\alpha\in R$. So $\delta'_{pk\alpha}(x)\leq 0$ for $x\in(0,\infty)$. A simple computation gives

$$\begin{aligned} \delta'_{pk\alpha}(x) =& \alpha x^{\alpha-1} \biggl[ \frac{1}{k}\ln \frac{pkx}{x+k(p+1)}-\psi_{pk}(x) \biggr] \\ & {}+x^{\alpha} \Biggl[\frac{p+1}{x(x+pk+k)}-\sum _{n=0}^{p}\frac {1}{(nk+x)^{2}} \Biggr]\leq0, \end{aligned}$$

which is equivalent to

$$\begin{aligned} &\alpha \biggl(\frac{1}{k}\ln\frac{pkx}{x+k(p+1)}-\psi _{pk}(x) \biggr) \\ &\quad {}+x \Biggl(\frac{p+1}{x(x+pk+k)}-\sum_{n=0}^{p} \frac {1}{(nk+x)^{2}} \Biggr)\leq0. \end{aligned}$$

Because of (see [17])

$$ \frac{1}{k}\ln\frac{pkx}{x+k(p+1)}-\psi_{pk}(x)>0, $$

we obtain

$$ \alpha\leq d(x)=\frac{x (-\frac{p+1}{x(x+pk+k)}+\sum_{n=0}^{p}\frac{1}{(nk+x)^{2}} )}{\frac{1}{k}\ln\frac {pkx}{x+k(p+1)}-\psi_{pk}(x)} $$

for all $x>0$.

Similarly as in [1], the proof will be done if we show that

$$ \lim_{x\rightarrow0^{+}}d(x)\leq1. $$

Direct computation leads to

$$\begin{aligned} \lim_{x\rightarrow0^{+}}d(x) &=\lim_{x\rightarrow0^{+}} \frac{-\frac{(p+1)x}{x+pk+k}+1+\sum_{n=1}^{p}\frac{x^{2}}{(nk+x)^{2}}}{x [\frac{1}{k}\ln\frac {pkx}{x+k(p+1)}-\frac{\ln(pk)}{k}+\frac{1}{x} +\sum_{n=1}^{p}\frac{1}{nk+x} ]} \\ &=\frac{1}{\lim_{x\rightarrow0^{+}}\frac{x}{k}\ln (\frac {x}{x+pk+k} )+1+\sum_{n=1}^{p}\frac{x}{nk+x}}=1. \end{aligned}$$

Indeed, $\lim_{x\rightarrow0^{+}}d(x)=1$ implies that, for each $\varepsilon>0$, there is $x_{\varepsilon}>0$ such that $d(x_{\varepsilon})<1+\varepsilon$, so $\alpha<1+\varepsilon$, and thus $\alpha\leq1$. This completes the proof.

### Disproving Conjecture 2

We show that Conjecture 2 is false. Let $0< a< b$. Put $y^{2}=a/b$. Then $0< y<1$. Conjecture 2 is equivalent to

$$ \biggl(1+\frac{1-y^{2}}{2\ln(y)} \biggr)\psi(b)- \biggl(\frac {1-y^{2}}{2\ln(y)}+y^{2} \biggr)\psi\bigl(by^{2}\bigr)> \bigl(1-y^{2} \bigr) \psi(by), $$

which can be rewritten as

$$ F(b,y)= \bigl(1-y^{2}+2\ln(y) \bigr) \bigl(\psi(b)-\psi \bigl(by^{2}\bigr) \bigr)-2\ln(y) \bigl(1-y^{2}\bigr) \bigl( \psi(by)-\psi\bigl(by^{2}\bigr) \bigr)< 0. $$

Let *b* be fixed. We prove that $\lim_{y\rightarrow 0^{+}}F(b,y)=+\infty$. This implies that Conjecture 2 does not valid. Using the well-known formula

$$ \psi(x)=-\gamma-\frac{1}{x}+\sum_{n=1}^{\infty} \frac{1}{n}-\frac{1}{n+x}, $$

we obtain

8 $$ \psi(b)-\psi\bigl(by^{2}\bigr)=\frac{1}{by^{2}}- \frac{1}{b}+\sum_{n=1}^{\infty} \frac{1}{n+by^{2}}-\frac{1}{n+b} $$

and

9 $$ \psi(by)-\psi\bigl(by^{2}\bigr)=\frac{1}{by^{2}}- \frac{1}{by}+\sum_{n=1}^{\infty} \frac{1}{n+by^{2}}-\frac{1}{n+by}. $$

So

$$\begin{aligned} F(b,y) =&\frac{1}{by^{2}} \bigl(1-y^{2}+2\ln(y) \bigr) \bigl(1-y^{2}+by^{2}\varphi_{1}(b,y) \bigr) \\ & {}-\frac{2\ln(y)(1-y^{2})}{by^{2}} \bigl(1-y+by^{2}\varphi _{2}(b,y) \bigr), \end{aligned}$$

where

$$ 0< \varphi_{1}(b,y)< \frac{b(1-y^{2})\pi^{2}}{6} \quad \text{and}\quad 0< \varphi_{2}(b,y)< \frac{by(1-y)\pi^{2}}{6}. $$

The function $F(b,y)$ may be rearranged as

$$\begin{aligned} F(b,y) =&\frac{1}{by^{2}}\bigl[\bigl(1-y^{2}\bigr) \bigl(1-y^{2}+by^{2}\varphi _{1}(b,y)\bigr) \\ &{}+ 2\ln(y) \bigl(by^{2}\varphi _{1}(b,y)+y-y^{3}-b \bigl(1-y^{2}\bigr)y^{2}\varphi_{2}(b,y) \bigr) \bigr]. \end{aligned}$$

This implies that $\lim_{y\rightarrow0^{+}}F(b,y)=+\infty$.

### Proof of Theorem 1

#### Theorem 1

*Let* $0< a< b<4/10$. *Then*

10 $$ \bigl(b-L(a, b)\bigr)\psi(b)+\bigl(L(a, b)-a\bigr)\psi(a)< (b-a)\psi (\sqrt{ba}). $$

#### Proof

It is easily derived that (10) is equivalent to $F(b,y)>0$, where $y^{2}=a/b$, $0< y<1$, and

$$\begin{aligned} F(b,y) =& \bigl(1-y^{2}+2\ln(y) \bigr) \bigl(\psi(b)-\psi \bigl(by^{2}\bigr) \bigr) \\ & {}-2\ln(y) \bigl(1-y^{2}\bigr) \bigl(\psi(by)-\psi \bigl(by^{2}\bigr) \bigr). \end{aligned}$$

Using (8), we obtain

$$\begin{aligned} \psi(b)-\psi\bigl(by^{2}\bigr) =&\frac{1-y^{2}}{by^{2}}+b \bigl(1-y^{2}\bigr)\sum_{n=1}^{\infty} \frac{1}{(n+by^{2})(n+b)} \\ < & \frac{1-y^{2}}{by^{2}}+b\bigl(1-y^{2}\bigr)\sum _{n=1}^{\infty}\frac{1}{(n+by)^{2}} \end{aligned}$$

due to $(n+by^{2})(n+b)>(n+by)^{2}$. So

11 $$ \psi(b)-\psi\bigl(by^{2}\bigr)< b\bigl(1-y^{2}\bigr) \psi'(by). $$

Applying (9), we get

$$\begin{aligned} \psi(by)-\psi\bigl(by^{2}\bigr) =&\frac{1-y}{by^{2}}+by(1-y)\sum _{n=1}^{\infty}\frac{1}{(n+by^{2})(n+by)} \\ >& \frac{1-y}{by^{2}}+b(1-y)\sum_{n=1}^{\infty} \frac{1}{(n+by)^{2}} \end{aligned}$$

due to $(n+by^{2})<(n+by)$. So

12 $$ \psi(by)-\psi\bigl(by^{2}\bigr)>\frac{1-y}{by^{2}} \bigl(1-y+b^{2}y^{3}\psi '(by) \bigr). $$

It is easy to see that, for $0< y<1$,

$$ s(y)=1-y^{2}+2\ln(y)< 0, $$

which follows from $s(1)=0$ and $s'(y)=2(1-y^{2})/y>0$. This implies that

$$\begin{aligned} F(b,y) >&G(b,y) \\ =&\frac{1}{b^{2}y^{2}}\bigl[\bigl(1-y^{2}+2\ln (y)\bigr)b \bigl(1-y^{2}\bigr)\psi'(by)) \\ & {}-2\ln(y) (1-y) \bigl(1-y^{2}\bigr) \bigl(1-y+b^{2}y^{3} \psi '(by) \bigr)\bigr]. \end{aligned}$$

The inequality $G>0$ is equivalent to

13 $$ b^{2}y^{2} \bigl(1-y^{2}+2\ln(y) \bigr) \psi'(by))-2\ln(y) (1-y) \bigl(1-y+b^{2}y^{3} \psi'(by) \bigr)>0. $$

Inequality (13) may be rearranged as

$$ H=\psi'(by)b^{2}y^{2} \bigl(1-y^{2}+2 \ln(y) \bigl(1-y+y^{2}\bigr) \bigr))-2\ln (y) (1-y)^{2}>0. $$

Put $s_{1}(y)=1-y^{2}+2\ln(y)(1-y+y^{2})$. It is easy to see that $s_{1}(y)<0$ for $0< y<1$. Indeed, $s_{1}(y)<0$ is equivalent to

$$ s_{2}(y)=\frac{y^{2}-1}{1-y+y^{2}}-2\ln(y)>0. $$

Due to $s_{2}(1)=0$, it suffices to show that $s'_{2}(y)<0$.

Differentiation leads to

$$ s'_{2}(y)=\frac{-2+3y-2y^{2}+3y^{3}-2y^{4}}{y(1-y+y^{2})^{2}}=\frac {-(1-y)^{2}(2+y+2y^{2})}{y(1-y+y^{2})^{2}}< 0. $$

Using the well-known formula

$$ \psi'(x)=\frac{1}{x^{2}}+\frac{1}{(1+x)^{2}}+\sum _{n=2}^{\infty }\frac{1}{(n+x)^{2}}, $$

we obtain

$$ b^{2}y^{2}\psi'(by)< 1+\frac{b^{2}y^{2}}{(1+by)^{2}}+b^{2}y^{2} \biggl(\frac{\pi^{2}}{6}-1 \biggr). $$

Theorem 1 will be proved if we show

$$\begin{aligned} G(b) =& \biggl(1+\frac{b^{2}y^{2}}{(1+by)^{2}}+b^{2}y^{2} \biggl( \frac {\pi^{2}}{6}-1 \biggr) \biggr) \bigl(1-y^{2}+2\ln(y) \bigl(1-y+y^{2}\bigr) \bigr) \\ &{} -2\ln(y) (1-y)^{2}>0 \end{aligned}$$

for $0< b<4/10$, $0< y<1$. Based on

$$ \frac{dG(b)}{db}= \bigl(1-y^{2}+2\ln(y) \bigl(1-y+y^{2} \bigr) \bigr) \biggl(\frac{2by^{2}}{(1+by)^{3}}+2by^{2} \biggl( \frac{\pi^{2}}{6}-1 \biggr) \biggr)< 0, $$

it suffices to prove that $G(0.4)>0$.

The inequality $G(b)>0$ is equivalent to

14 $$ 2\ln(y)f(b,y)+g(b,y)>0, $$

where

$$\begin{aligned}& \begin{aligned} f(b,y)&=\bigl(1-y+y^{2}\bigr) \biggl[(1+by)^{2}+b^{2}y^{2} \biggl(1+(1+by)^{2} \biggl(\frac{\pi^{2}}{6}-1 \biggr) \biggr) \biggr] \\ &\quad {}-(1+by)^{2}(1-y)^{2}, \end{aligned} \\& g(b,y)=\bigl(1-y^{2}\bigr) \biggl[(1+by)^{2}+b^{2}y^{2} \biggl(1+(1+by)^{2} \biggl(\frac{\pi^{2}}{6}-1 \biggr) \biggr) \biggr]. \end{aligned}$$

It is clearly seen that $f(b,y)>0$. So (14) will be done if we prove

$$ h(b,y)=2\ln(y)+\frac{g(b,y)}{f(b,y)}>0 $$

for $b=4/10$ and $0< y<1$. Because of $h(0.4,1)=0$, it suffices to show that $(dh/dy)(0.4,y)<0$ for $0< y<1$. We get

15 $$ \frac{dh(0.4,y)}{dy}=\frac{2}{y}+\frac{\frac {dg(0.4,y)}{dy}f(0.4,y)-g(0.4,y)\frac{df(0.4,y)}{dy}}{f^{2}(0.4,y)}< 0. $$

Inequality (15) is equivalent to

16 $$ u(y)=2f^{2}(0.4,y)+y \biggl(\frac{dg(0.4,y)}{dy}f(0.4,y)-g(0.4,y) \frac {df(0.4,y)}{dy} \biggr)< 0. $$

Put $a(y)=100u(y)$. Using Taylor’s series and Matlab, we obtain

$$\begin{aligned} a(y) =& (y-1)^{2} \bigl(899.80856587904507327359937090692\,(y-1) \\ &{}- 34.541951843593497556069703611525 \\ &{}+ 2803.0064998600206956003380918157(y-1)^{2} \\ &{} + 3449.9649390326664508411882274417(y-1)^{3} \\ &{} + 2382.8365919732490773391558060343(y-1)^{4} \\ &{} + 1077.4495988779774279297213875464(y-1)^{5} \\ &{} + 341.35184858325869449609722858626(y-1)^{6} \\ &{} + 75.928581022558019892100963581518(y-1)^{7} \\ &{} + 11.561798822762785643694391575539(y-1)^{8} \\ &{}+ 1.1176206646533878730984733813133(y-1)^{9} \\ &{} + 0.05451808120260428649260845762504(y-1)^{10}\bigr) ) \\ =&(y-1)^{2}k(y), \end{aligned}$$

where

$$\begin{aligned} k(y) =& 899.80856587904507327359937090692(y-1) \\ &{} -34.541951843593497556069703611525 \\ &{} +2803.0064998600206956003380918157(y-1)^{2} \\ &{} +3449.9649390326664508411882274417(y-1)^{3} \\ &{} +2382.8365919732490773391558060343(y-1)^{4} \\ &{} +1077.4495988779774279297213875464(y-1)^{5} \\ &{} +341.35184858325869449609722858626(y-1)^{6} \\ &{} +75.928581022558019892100963581518(y-1)^{7} \\ &{} +11.561798822762785643694391575539(y-1)^{8} \\ &{} +1.1176206646533878730984733813133(y-1)^{9} \\ &{} +0.05451808120260428649260845762504(y-1)^{10}. \end{aligned}$$

It is easy to see that $k(y)< kk(y)$, where

$$\begin{aligned} kk(y) =& 899.80856587904507327359937090692(y-1) \\ &{} -34.541951843593497556069703611525 \\ &{} +2803.0064998600206956003380918157(y-1)^{2} \\ &{} +3449.9649390326664508411882274417(y-1)^{3} \\ &{} +2382.8365919732490773391558060343(y-1)^{4} \\ &{} +1077.4495988779774279297213875464(y-1)^{5} \\ &{} +341.35184858325869449609722858626(y-1)^{6} \\ &{} +75.928581022558019892100963581518(y-1)^{7} \\ &{} +11.561798822762785643694391575539(y-1)^{8} \\ &{} +1.1176206646533878730984733813133(y-1)^{9} \\ &{} +0.05451808120260428649260845762504(y-1)^{6}. \end{aligned}$$

To prove that $kk(y)<0$, it suffices to show that $kk(0)\leq0$, $kk(1)\leq0$, $kk'(0)\leq0$, $kk''(1)>0$, $kk''(0)<0$, and $kk''(y)$ is an increasing function on $(0,1)$.

Put $c(y)=kk''(y)$. Direct computation yields

$$\begin{aligned} c(y) =& (22\text{,}759\text{,}659\text{,}395\text{,}315\text{,}613y)/1 \text{,}099\text{,}511\text{,}627\text{,}776 \\ &{} +\bigl(2\text{,}021\text{,}889\text{,}693\text{,}856\text{,}018 \text{,}983(y-1)^{2}\bigr)/70\text{,}368\text{,}744\text{,}177 \text{,}664 \\ &{} +\bigl(23\text{,}693\text{,}367\text{,}246\text{,}178\text{,}465(y-1)^{3} \bigr)/1\text{,}099\text{,}511\text{,}627\text{,}776 \\ &{} +\bigl(738\text{,}027\text{,}641\text{,}132\text{,}151\text{,}741 \text{,}645(y-1)^{4}\bigr)/72\text{,}057\text{,}594\text{,}037 \text{,}927\text{,}936 \\ &{} +\bigl(112\text{,}202\text{,}976\text{,}768\text{,}737 \text{,}799(y-1)^{5}\bigr)/35\text{,}184\text{,}372\text{,}088 \text{,}832 \\ &{} +\bigl(11\text{,}324\text{,}961\text{,}019\text{,}967\text{,}769(y-1)^{7} \bigr)/140\text{,}737\text{,}488\text{,}355\text{,}328 \\ &{} -8\text{,}297\text{,}891\text{,}458\text{,}330\text{,}007/549\text{,}755 \text{,}813\text{,}888. \end{aligned}$$

We now show that $cc(y)=kk'''(y)>0$. We have

$$\begin{aligned} cc(y) =& 57\text{,}465.561379104286260144363041036y \\ &{} +64\text{,}646.975932678647041029762476683(y-1)^{2} \\ &{} +40\text{,}968.763999735354855158409037585(y-1)^{3} \\ &{} +15\text{,}945.002014737184339310260838829(y-1)^{4} \\ &{} +563.28081498530752213582672993653(y-1)^{6} \\ &{} -36\text{,}765.771744908288582109889830463. \end{aligned}$$

Differentiation yields

$$\begin{aligned} cc''(y) =& 16\text{,}898.424449559224740369245409966y^{4} \\ &{} -67\text{,}593.697798236898961476981639862y^{3} \\ &{} +292\text{,}730.57087420154857682064175606y^{2} \\ &{} -204\text{,}461.16215351717255543917417526y \\ &{} +91\text{,}719.816493350586824817582964897. \end{aligned}$$

Using the Cardano formula and Matlab, we get that there are no real roots of $cc''(y)=0$. Due to $cc(0)>0$, we obtain $cc''(y)>0$.

We now show that $v(y)>0$ for $0< y<1$, where $v(y)$ is a tangent line to the function $cc(y)$ at the point $(0.22,cc(0.22))$.

Using Matlab, we have

$$cc(0.22)=1794.965061908937 \quad \mbox{and}\quad cc'(0.22)=149.7626334452943. $$

This implies that

$$ v(y)=1794.965061908937+149.7626334452943(y-0.22). $$

Direct computation yields:

$$\begin{aligned}& v(0)=1762.017282550972 , \qquad v(1)=1911.779915996267 , \\& kk(0)=-2.3294\mathrm{e}{-}14 , \qquad kk(1)=-34.5420 , \qquad kk'(0)=-53.5347 , \\& kk''(0)=-937.2660 , \qquad kk''(1)=5.6060\mathrm{e}{+}03 . \end{aligned}$$

This completes the proof. □

### Open problem

Finally, we give an open problem.

#### Open Problem 2

Find the best possible real positive constants $b_{0}$, $b_{1}$ such that if $0< a< b\leq b_{0}$, then

$$ \bigl(b-L(a, b)\bigr)\psi(b)+\bigl(L(a, b)-a\bigr)\psi(a)< (b-a)\psi (\sqrt{ba}), $$

and if $0< b_{1}\leq a< b$, then

$$ \bigl(b-L(a, b)\bigr)\psi(b)+\bigl(L(a, b)-a\bigr)\psi(a)>(b-a)\psi (\sqrt{ba}), $$

where $L(a, b)=(b-a)/(\ln(b)-\ln(a))$.

#### Note 1

Note that our work and [3] show that $4/10\leq b_{0}$ and $2\geq b_{1}$.

## Conclusion

In this paper, we proved e Open Problem 1 [17] and disproved Conjectures 1 and 2 [3, 19]. We also proved a new inequality (Theorem 1) for the digamma function. Finally, we proposed an Open Problem 2.
